# Poly(ADP-ribose) polymerase-1 polymorphisms, expression and activity in selected human tumour cell lines

**DOI:** 10.1038/sj.bjc.6605166

**Published:** 2009-06-30

**Authors:** T Zaremba, P Ketzer, M Cole, S Coulthard, E R Plummer, N J Curtin

**Affiliations:** 1 Northern Institute for Cancer Research, Newcastle University, Paul O'Gorman Building, Newcastle upon Tyne NE2 4HH, UK; 2 Department of Biology, University of Konstanz, Konstanz 78457, Germany

**Keywords:** PARP, PARP activity, PARP expression, polymorphisms, cancer cell lines

## Abstract

**Background:**

Poly(ADP-ribose) polymerase-1 (PARP-1) is a DNA-binding enzyme activated by DNA breaks and involved in DNA repair and other cellular processes. Poly(ADP-ribose) polymerase activity can be higher in cancer than in adjacent normal tissue, but cancer predisposition is reported to be greater in individuals with a single-nucleotide polymorphism (SNP) V762A (T2444C) in the catalytic domain that reduces PARP-1 activity.

**Methods:**

To resolve these divergent observations, we determined *PARP-1* polymorphisms, PARP-1 protein expression and activity in a panel of 19 solid and haematological, adult and paediatric human cancer cell lines.

**Results:**

There was a wide variation in PARP activity in the cell line panel (coefficient of variation, CV=103%), with the lowest and the highest activity being 2460 pmol PAR/10^6^ (HS-5 cells) and 85 750 pmol PAR/10^6^ (NGP cells). Lower variation (CV=32%) was observed in PARP-1 protein expression with the lowest expression being 2.0 ng *μ*g^−1^ (HS-5 cells) and the highest being 7.1 ng *μ*g^−1^ (ML-1 cells). The mean activity in the cancer cells was 45-fold higher than the mean activity in normal human lymphocytes and the PARP-1 protein levels were 23-fold higher.

**Conclusions:**

Surprisingly, there was no significant correlation between PARP activity and PARP-1 protein level or the investigated polymorphisms, T2444C and CA.

Poly(ADP-ribose) polymerase-1 (PARP-1) is a DNA repair enzyme that catalyses the poly(ADP-ribosyl)ation of proteins using NAD^+^ as its substrate. Poly(ADP-ribose) polymerase-1 is activated 100-fold by DNA strand breaks and has a major role in DNA single-strand break repair (which is part of base excision repair) (Dantzer *et al*, 1999, 2000). Recent data also suggest that PARP-1 is involved in DNA double-strand break (DSB) repair (von Kobbe *et al*, 2003); however, its role in DSB repair remains unclear. As a result of compromised repair, PARP-1 deficient or inhibited cells are more sensitive to DNA-damaging agents (*γ*-irradiation, topoisomerase inhibitors, alkylating agents) (Curtin, 2005). Poly(ADP-ribose) polymerase-1 has an important function in the fine-tuning of telomere length (Beneke *et al*, 2008) and, together with other PARP-family members, namely PARP-3, PARP-4 and PARP-5, is present in mitotic apparatus and therefore might be involved in the regulation of chromosomal separation (Miwa *et al*, 2006). As chromosomal missegregation and centrosome amplification frequently occur in cancer cells, leading to the characteristic aneuploidy, PARP-1 may protect against carcinogenesis. Poly(ADP-ribose) polymerase-1 also has an important function in cell-cycle control, especially after DNA damage (Nozaki *et al*, 1994). Poly(ADP-ribose) polymerase-1 clearly has the potential to inhibit the development of cancer, by promoting genomic stability through DNA repair and cell-cycle control. Consistent with this view is the finding that individuals with a polymorphism that confers reduced PARP activity have increased risk of cancer (see below). In addition, despite a general decline in PARP activity with age (Grube and Bürkle, 1992; Chevanne *et al*, 2007) that may correspond to the increase in cancer incidence with age (http://info.cancerresearchuk.org/cancerstats/mortality/age/), lymphocytes from people who live to a very old age (>100 years) without developing cancer have higher PARP activity (Muiras *et al*, 1998). With regard to the association of PARP-1 polymorphisms with activity and cancer, the T2444C single-nucleotide polymorphism (SNP; Cottet *et al*, 2000) results in an amino-acid substitution, Val762Ala, in the PARP-1 activity domain. The loss of a methyl group from Val moves the 762 residue further away from the G888 residue (from 4.01 Å for valine to 5.19 Å for alanine), which is located in the conserved during evolution region of the active site called ‘PARP signature’ and crucial for enzyme activity (Cottet *et al*, 2000). It has been reported that T2444C SNP reduces PARP-1 catalytic activity by 30–40% (Lockett *et al*, 2004; Wang *et al*, 2007). The variant form was found to be associated with prostate cancer (two-fold increase in susceptibility), oesophageal and lung cancer in Chinese smokers and thyroid carcinoma (Hao *et al*, 2004; Lockett *et al*, 2004; Zhang *et al*, 2005; Chiang *et al*, 2008). Other factors may also influence PARP-1 activity, such as PARP-1 expression. Polymorphisms in the promoter region of *PARP-1* gene may influence PARP-1 protein expression. A microsatellite polymorphism in the *PARP-1* promoter, consisting of a variable number of CA repeats has been identified (Fougerousse *et al*, 1992). Long microsatellite (CA)_n_ repeats may facilitate the formation of DNA racket structures in the promoter region that bring the TATA boxes into vicinity of the transcription factor-binding site (Schweiger *et al*, 1995). Furthermore, the (CA)_n_ microsatellite is located close to the binding site of the transcription factor Yin Yang 1(YY1), and this may contribute to the regulation of PARP-1 expression (Oei and Shi, 2001).

In contrast to the proposed role of PARP-1 activity in inhibiting cancer development, PARP expression and/or activity is generally higher in cancer tissue compared with normal tissue. A higher level of PARP-1 mRNA was observed in malignant lymphoma cells compared with normal lymph nodes (Tomoda *et al*, 1991) and in colorectal adenocarcinoma biopsies compared with adjacent non-tumour tissues and hyperplastic polyps (Nosho *et al*, 2006). Evaluation of PARP-1 protein expression in hepatocellular carcinoma (HCC) by western blotting revealed that it was significantly increased in HCC, especially moderately differentiated, compared with the non-tumour samples (Shimizu *et al*, 2004). Increased poly(ADP-ribosyl)ation has also been reported in cancers compared with adjacent tissues, i.e., HCC (Nomura *et al*, 2000), colon carcinoma (Hirai *et al*, 1983), cervical cancer (Fukushima *et al*, 1981), melanoma and basal cell carcinoma (Ikai *et al*, 1980). Recent studies link PARP-1 to inflammation and cancer through the activation of the stress-inducible transcription complex, NF-*κ*B. Nuclear factor-*κ*B inhibits apoptosis, stimulates proliferation and synthesis of proinflammatory mediators, critical components of tumour progression (Coussens and Werb, 2002). Nuclear factor-*κ*B activity is elevated in a wide spectrum of cancers and is correlated with malignancy and progression (Rayet and Gelinas, 1999). Poly(ADP-ribose) polymerase-1 was found to be essential for NF-*κ*B transcriptional activation (Hassa and Hottiger, 1999; Oliver *et al*, 1999) and the regulation of IR-induced NF-*κ*B activation (Veuger *et al*, 2009).

Because of these somewhat opposing observations, that polymorphisms associated with reduced PARP-1 activity are associated with cancer predisposition on the one hand, and increased PARP activity in tumours on the other, we set out to investigate PARP-1 polymorphisms, expression and activity, using assays validated to GCLP for clinical trials, in a panel of 19 human cancer cell lines and one human immortalised cell line. As the majority of reported studies on human PARP-1 polymorphisms and activity have been conducted using lymphocytes, half of our panel were of leukaemic/lymphatic origin and included the immortalised human bone marrow stromal cells. The remaining cells were from solid tumours representing common paediatric tumours (a panel of five neuroblastoma cell lines – the most common extracranial solid tumour) and adult tumours (three breast cancer cell lines with different hormone receptor status and one colorectal cell line) as well as a rarer type (thyroid). We aimed to correlate PARP-1 protein expression with the length of the (CA)_n_ promoter microsatellite and PARP activity with PARP-1 protein levels and the T2444C SNP. We found a significantly higher frequency of the T2444C polymorphism than that reported in the general population, higher levels of PARP expression and activity than those reported for normal human lymphocytes, and a high variation in PARP activity (coefficient of variation, CV=103%) and a low variation in expression (CV=32%) between the cell lines. Unexpectedly, there was no correlation of PARP activity with PARP-1 protein expression or the polymorphisms.

## Materials and methods

Pre B697 were a generous gift from Prof R Kofler (Innsbruck, Austria). We obtained all other cell lines from ECACC (Salisbury, Wilts, UK) or ATCC (Manassas VA, USA). Studies were conducted using five neuroblastoma cell lines (TR14, NB1691, NGP, LS, SK-N-BE2C), three breast cancer cell lines (T47D, MCF-7, MDA-MB-231), eight leukaemia cell lines (Nalm6, Pre B697, HL-60, K562, CCRF-CEM, Jurkat, Molt-4, TK-6), one Burkitt's lymphoma cell line (Raji), thyroid carcinoma cell line (ML-1) and colorectal carcinoma cell line (LoVo), respectively. We also examined immortalised bone marrow stromal cell line (HS-5). All cells were grown in RPMI medium with 10% fetal bovine serum at 37°C in an atmosphere of 5% CO_2_ in air. Cells were confirmed mycoplasma negative by regular testing (Mycoalert; Cambrex, Charles City, IA, USA). All chemicals and reagents were of the highest quality and supplied by Sigma (Dorset, UK), unless otherwise stated. The PARP inhibitor AG014699 was supplied by Pfizer GRD (La Jolla, CA, USA). The 10H mouse monoclonal primary antibody was generously provided by Prof Alexander Bürkle (University of Konstanz, Germany).

### Measurement of PARP-1 activity

We measured PARP activity by modification of a previously described method (Plummer *et al*, 2005) validated to GCLP standard and used as a pharmacodynamic end point for clinical trials (Plummer *et al*, 2008). Briefly, maximally stimulated PARP activity was measured in triplicate samples of 2500 cells permeabilised with digitonin (Sigma, Dorset, UK) in a reaction mixture containing 350 *μ*mol l^−1^ NAD^+^ as substrate and 10 *μ*g ml^−1^ PARP-1 activating oligonucleotide (CGGAATTCCG) (Europrim, Invitrogen, Cambridge, UK) in a reaction buffer of 100 mmol l^−1^ Tris-HCl, 120 mmol l^−1^ MgCl_2_ (pH 7.8) in a final volume of 100 *μ*l at 26°C in an oscillating water bath. The reaction was stopped after 6 min by the addition of excess PARP inhibitor (400 *μ*l of 12.5 *μ*mol l^−1^ AG014699) and the cells were blotted along with a poly(ADP-ribose) standard (Biomol, Exeter, UK) onto a nitrocellulose membrane (Hybond-N, Amersham, Little Chalford, UK) using a purpose-built 48-well manifold. After an overnight incubation with the primary anti-PAR 10H antibody (1 : 500 in phosphate-buffer solution (PBS) containing 5% milk (fat free) and Tween-20 (PBS-MT)) at 4°C, two washes in PBS-T, followed by incubation in HRP-conjugated goat anti-mouse secondary antibody (1 : 1000 in PBS-MT; Dako) for 1 h at room temperature, and further frequent washes with PBS for 1 h, the membrane was exposed for 1 min to an enhanced chemiluminescence (ECL) reaction solution (Amersham), according to the manufacturer's instructions; chemiluminesence was measured during a 5-min exposure using a Fuji LAS3000 with imaging software (Fuji LAS Image version 1.1, Raytek, Raytek Scientific, Sheffield, UK) and analysed using Aida Image Analyzer software (version 3.28.001, Raytek Scientific, Sheffield, UK). Results were expressed in LAU/mm^2^ relative to the number of cells loaded and subsequently calculated by reference to the poly(ADP-ribose) standard curve (0–25 pmol).

### Western blot analysis

Briefly, we prepared cell lysates by adding 100 *μ*l of Laemmli buffer (Laemmli, 1970) with protease inhibitor cocktail (Thermo Fisher Scientific, Rockford, IL, USA) to the cell pellet, resuspending by pipetting and leaving on ice for 30 min with a brief mix every 5 min before sonication on ice for 10 s using 20 *μ* amplitude (Vibracell Sonicator, Sonics and Materials, Danbury, CT, USA) and heating in loading dye containing *β*-mercaptoethanol and bromophenol blue at 95°C for 5 min. Lysates (5 *μ*g of protein per lane) were loaded onto Tris-HCl 5–20% polyacrylamide gels (Invitrogen, Glasgow, UK) along with PARP-1 immunoblotting standard and after.

Electrophoresis at 100 V for 2 h (Criterion electrophoresis apparatus, Bio-Rad, Hercules, CA, USA), the proteins were transferred for 1 h at 4°C into a nitrocellulose membrane (Hybond-C, Amersham) using a Criterion Blotter (Bio-Rad). After blocking for 1 h in PBS containing 5% milk (fat free) and Tween-20 (PBS-MT), the membrane was incubated overnight at 4°C with shaking with an anti-PARP-1 C2-10 primary antibody (Trevigen, Gaithersburg, MD, USA) diluted 1 : 2000 in (PBS-MT), washed 3 times for 15 min in PBS/Tween-20 (PBS-T), and then incubated with the HRP-linked secondary goat anti-mouse antibody (Dako, Ely, UK) diluted 1 : 1000 in PBS-MT, washed again for 1 h in PBS-T with changing buffer every 5 min and then dried. The protein was visualised with the ECL plus detection kit (Amersham) using the manufacturer's protocol followed by chemiluminescence detection as described above. We quantified the PARP-1 expression by reference to purified recombinant PARP-1 protein (Biomol) standard curve (0–40 ng). This assay has also been validated to GCLP standard for evaluation of patient samples (E Mulligan and T Zaremba, unpublished data).

### T2444C genotyping

We isolated DNA directly from the cell lines using a Blood Mini Kit (Qiagen, Crawley, West Sussex, UK) according to the manufacturer's instructions and then genotyped 50 ng of genomic DNA by pyrosequencing using the PSQ96 system (Pyrosequencing, Uppsala, Sweden) as described earlier (Alderborn *et al*, 2000). Briefly, we amplified the genomic fragment containing the SNP site by PCR with a set of sense and antisense primers (5′-AGTCTGTCTCATTCACCAT-3′ and 5′-ATCCTTGCTGCTATCATC-3′) where antisense was 5′-biotinylated (Europrim, Invitrogen). We purified the PCR products using streptavidin-modified paramagnetic beads (Dynal, Skoyen, Norway) and determined the nucleotide sequence by pyrosequencing chemistry of the denatured product.

### Microsatellite (CA)_n_ repeats

To determine PARP-1 CA microsatellite genotyping we used standard protocols and touchdown PCR for amplification, with annealing temperature of 50°C and previously published sense and antisense primers (5′-GATTCCCCATCTCTTTCTTT-3′ and 5′-AAATTGTGGTAATGACTGCA-3′). The sense primer was 5′-labelled with the WellRed Beckman fluorescent dye D4 (Proligo, Paris, France). We added 1 *μ*l aliquots of the PCR product to 30 *μ*l of formamide and 0.3 *μ*l internal size standard (Beckman Coulter, High Wycombe, Buckinghamshire, UK) and analysed them in denaturating gels (Beckman Coulter) by capillary electrophoresis system CEQ8000 (Beckman Coulter).

### Statistical analysis

We analysed each sample in triplicate in three independent experiments and expressed the data as the mean value ±s.d. We applied a base-10 logarithmic transformation to PARP activity and expression. We examined the association between PARP activity and expression by Pearson's correlation analysis using GraphPad Prism4 software (GraphPad, La Jolla, CA, USA). We calculated *P*-values for genotype frequencies analysis by the Freeman–Halton extension of Fisher's exact test (VassarStats, online software, Poughkeepsie, NY, US) and for difference in PARP-1 expression and activity between the cell lines by Student's *t*-test (GraphPad).

## Results

### PARP active site SNP T2444C (Val762Ala)

Analysis of the T2444C (Val762Ala) SNP in all cancer cell lines revealed that most of the cell lines have the wild genotype T/T (80%). The leukaemia cell line, Pre B697, and the colorectal carcinoma cell line, LoVo, have a heterozygote genotype (T/C), whereas LS and Jurkat cells have a homozygote SNP (C/C) (Table 1). Analysis of the frequencies of the active site T2444C SNP showed that the frequency of the variant allele is significantly higher (*P*=0.02; Freeman–Halton extension of Fisher's exact test) than in the general Caucasian population (73% T/T, 25% T/C, 2% C/C; Lockett *et al*, 2004).

### (CA)_n_ microsatellite instability in PARP-1 promoter region

It has been proposed that the long CA polymorphism in the promoter region of *PARP-1* gene may result in an increased PARP-1 expression. We therefore investigated the length of this microsatellite repeat sequence in our cell line panel. As established earlier (Pascual *et al*, 2003), we grouped the CA microsatellite into two alleles:

S (short)−(CA)_11−12_ and L (long)−(CA)_13−20_. We found the following genotype frequencies: 61% SS (TK-6, HS-5, MDA-MB-231, MCF-7, Pre B697, Naml6, CCRF-CEM, K562, TR14, NB1691, Jurkat), 28% SL (ML-1, T47D, Molt-4, HL-60, SK-N-BE2C), 11% LL (Raji, LoVo) (Table 1). It was not possible to establish the CA repeat length for LS and NGP cells. Analysis of the length of the CA microsatellite revealed that most of the cell lines have short repeats, and the genotype frequencies are not significantly different (Freeman–Halton extension of Fisher's exact test; *P*=0.6) from those in PBMC from healthy volunteers (60% were SS, 20% SL and 20% LL) (Zaremba *et al*, 2008).

### PARP-1 expression

**Figure 1:**
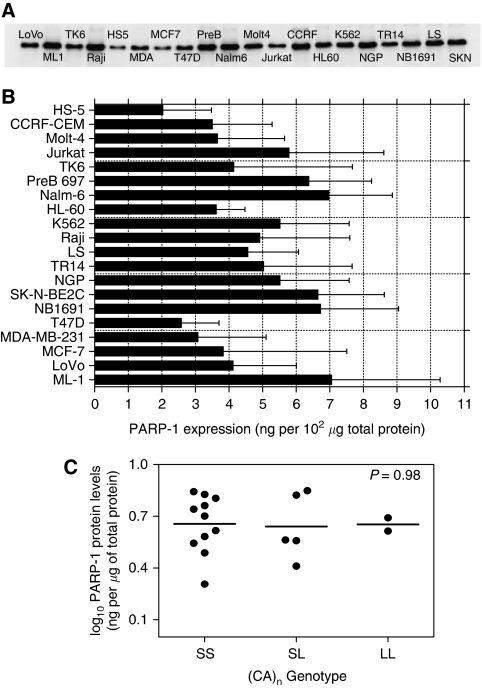
PARP-1 expression in the cell line panel. (**A**) Upregulation of PARP-1 expression in tumour cell lines detected by western blot analysis. (**B**) Semiquantitative analysis of the results of replicate experiments of the kind shown in **A**: Columns, mean of samples analysed in triplicate in three separate experiments; bars, s.d.; line, mean of all samples. (**C**) PARP-1 protein levels (expressed as log_10_) in relation to (CA)_n_ microsatellite polymorphisms; SS – short alleles (CA)_11–12_/(CA)_11–12,_ SL – short/long alleles (CA)_11–12_/(CA)_13–20_, LL – long/long alleles (CA)_13–20_/(CA)_13–20._ *P*-value calculated by Student's *t*-test (SS *vs* SL).

We measured the PARP-1 protein expression in the cell lines by semiquantitative western blot (Figure 1A). Analysis of pooled data from replicate experiments revealed a 3.5-fold difference in the PARP-1 expression level between the lowest (2.0 ng *μ*g^−1^ protein for HS5 cells) and the highest (7.1 ng *μ*g^−1^ protein for ML-1 cells) with the mean PARP-1 expression being 4.8±1.5 ng *μ*g^−1^ protein (mean±s.d.) (Figure 1B) and CV=32%. Two breast cancer cell lines (T47D, MDA-MB-231) and the bone marrow stromal cell line (HS-5) showed lower expression levels of PARP-1 compared with the other cell lines (<mean−s.d.), whereas two neuroblastoma cell lines (NB 1691, SK-N-BE2C), two leukaemia cell lines (Nalm6, PreB) and thyroid carcinoma cell line (ML-1) showed higher expression levels (>mean+s.d.). We did not find any significant association between the level of PARP-1 expression and the length of CA repeats (SS *vs* SL; *P*=0.98; Student's *t*-test) (Figure 1C).

### PARP activity

**Figure 2:**
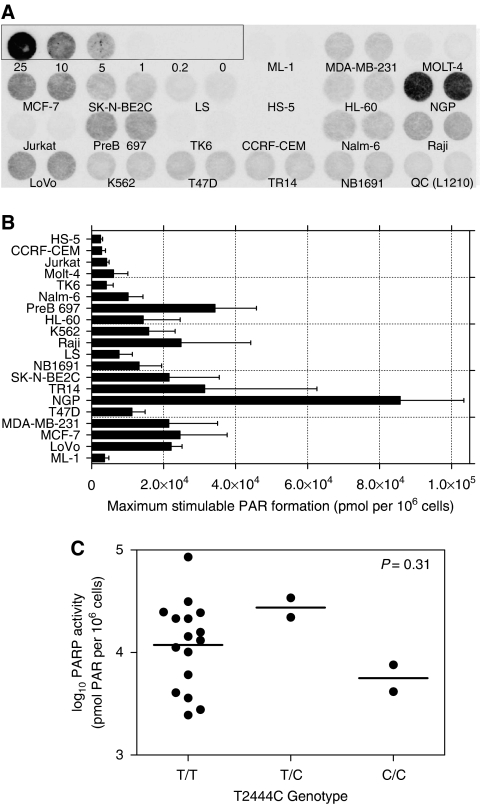
PARP activity in the cell line panel. (**A**) PAR formation detected by immunoblot assay. (**B**) Semiquantitative analysis of the results of replicate experiments of the kind shown in **A**: Columns, mean of samples analysed in triplicate in three separate experiments; bars, s.d. (**C**) PARP activity (expressed as log_10_) in individuals with 2444 TT, TC, and CC genotype, respectively. *P*-value calculated by Student's *t*-test (T/T *vs* C/C).

**Figure 3:**
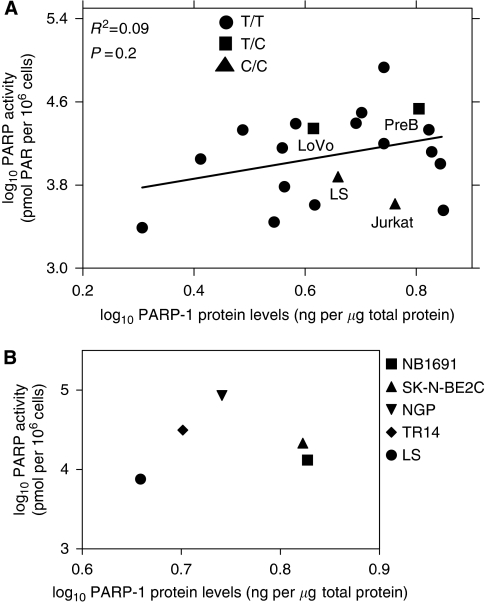
Correlation between PARP-1 protein levels and PARP activity. (**A**) On the graph cell lines with variant genotype for T2444C SNP are indicated by squares and triangles. (**B**) Correlation between PARP-1 expression and activity in five neuroblastoma cell lines.

Measurement of PARP activity in the cell line panel revealed a wide variation between the cell lines (CV=103%). HS5 cells had the lowest activity (2460 pmol PAR/10^6^ cells) and the NGP cells had the highest activity (85 750 pmol PAR/10^6^ cells) with the mean activity across the panel being 18 069±18 679 pmol PAR/10^6^ cells (mean±s.d.) (Figure 2B). This variation is significantly greater than the PARP-1 expression (CV=32%). To see if PARP activity was dependent on the level of protein expression, we compared the activity and expression data (Figure 3). Pearson's correlation analysis showed that there was no significant correlation between activity and expression (*R*^2^=0.09, *P*=0.2) (Figure 3).

## Discussion

By measuring selected polymorphisms in the *PARP-1* gene as well as PARP-1 protein levels and activity in a panel of human cancer cell lines, we hoped to uncover new evidence of the relationship between genotype, expression and activity and also to gain further understanding of the relationship between PARP-1 and cancer formation. Analysis of the genotype frequencies of the active site T2444C SNP (80% T/T, 10% T/C and 10% C/C) revealed that the frequency of the variant allele is significantly higher than in the general population (Freeman–Halton extension of Fisher's exact test; *P*=0.02). This is consistent with earlier studies indicating that individuals with the variant polymorphism are at higher risk of developing cancer (Hao *et al*, 2004; Lockett *et al*, 2004; Zhang *et al*, 2005). However, although the variant polymorphism was associated with reduced PARP-1 activity in normal cells, this was not the case in our panel of cancer cells. Although the LS neuroblastoma cells with the C/C genotype had the lowest PARP activity among all analysed neuroblastoma cell lines and Jurkat (leukaemic) cells also had the C/C SNP and low PARP activity, four other cell lines with very low PARP activity, namely, two leukaemia cell lines CCRF-CEM and TK6, thyroid carcionoma cell line ML-1 and immortalised bone marrow stromal cells HS-5, did not possess variant allele of T2444C. Therefore, due to the small sample number, we did not find any significant correlation between PARP-1 activity and T2444C genotype (Figure 2C).

The length of the CA microsatellite in the promoter has not only been implicated in transcription of PARP-1 but has also been described as the haplotype defining variant of the whole *PARP-1* promoter polymorphism (Pascual *et al*, 2003). Previous studies showed that a long CA microsatellite is related to autoimmune diseases, namely, rheumatoid arthritis and coeliac disease (Pascual *et al*, 2003; Rueda *et al*, 2005). This evidence implicates PARP-1 primarily in inflammatory processes leading to the development of immunological disorders, but these may also lead to promotion and development of cancer by NF-*κ*B dependent or independent mechanisms. Our study is the first investigation of a role for the CA microsatellite in PARP-1 expression in cancer cells. Studies on the length of the CA microsatellite revealed that most of the cell lines have short repeats, and the genotype frequencies are not significantly different (*P*=0.6; Freeman–Halton extension of Fisher's exact test) from those in PBMC from healthy volunteers (Zaremba *et al*, 2008). Overall, our data show significant upregulation (approximately 23-fold higher) of PARP-1 expression in the cancer cell lines compared with human lymphocytes from healthy volunteers and cancer patients (Zaremba *et al*, 2008). Our finding is in accordance with earlier studies showing increased expression of PARP-1 in cancer, as described in the Introduction section. Poly(ADP-ribose) polymerase-1 protein expression varied between the cell lines; however, we did not find any correlation between the level of PARP-1 expression and length of the CA-repeats (Figure 1C).

We found that on average the cell lines had a 45-fold higher activity than the mean activity measured in PBMC from healthy volunteers and cancer patients (403.5±617.3 pmol PAR/10^6^ cells; Zaremba *et al*, 2008). This finding is in accordance with earlier studies described in the Introduction section showing increased PARP activity in tumours. There was a large variation in PARP-1 activity between different cell lines (CV=103%), which was much greater than the variation in expression (CV=32%). Furthermore, our results show that there is no statistically significant positive correlation between PARP-1 expression and PARP-1 activity in the panel overall (Figure 3A) or when comparing cells from the same tissue of origin, e.g., the five neuroblastoma cell lines (Figure 3B). This contrasts to earlier data showing correlation of PARP activity with PARP-1 protein expression in five colon cancer cell lines (Tentori *et al*, 2006). The lower than expected activity in cells with high levels of expression (e.g., ML-1) could not be explained by the T2444C SNP and indeed the cells with the variant allele did not appear to have lower activity than expected on the basis of expression (Table 1). Interestingly, the immortalised human marrow stromal cells, HS-5, had the lowest PARP-1 protein concentration and the lowest PARP activity, suggesting that the super-high levels of expression and activity may be specifically associated with malignant progression.

Our data show that PARP activity is not determined by the level of the enzyme and/or a polymorphism in the DNA sequence encoding the enzyme active site. This suggests that PARP-1 activity is regulated by either posttranslational modification and/or endogenous activation or repression. The assay we used in this study, by the use of an oligonucleotide, measures total stimulatable PARP activity and therefore cellular events that might activate PARP-1 endogenously, such as oxidative DNA damage, will not contribute to the variation in activity observed. There are numerous factors that might have an impact on PARP-1 activity; DNA-PK may activate PARP-1 by phosphorylation (Ruscetti *et al*, 1998) or suppress PARP activity through sequestration of DNA ends that serve as an important stimulator for both enzymes (Ariumi *et al*, 1999). Inactive DNA-PK has also been shown to suppress the activity of PARP-1, an effect that was not due to substrate competition, as DNA ends were provided in excess (Veuger *et al*, 2004). ERK1/2 kinase has also been reported to phosphorylate and activate PARP-1 (Kauppinen *et al*, 2006). There is a strong evidence in the literature showing that PARP expression and activity are affected by differentiation and cell proliferation with generally higher PARP activity and expression in proliferating cells (Wein *et al*, 1993). Negroni and Bertazzoni (1993) showed that growing HeLa cells and mitogen-stimulated human lymphocytes have very similar levels of PARP-1 mRNA, whereas in quiescent lymphocytes the value was 20-fold lower. More recently, Carbone *et al* (2008) showed that PARP-1 activity was induced by mitogen stimulation and contributed to the G0–G1 cell-cycle transition by the induction of immediate-early genes such as c-Myc and c-Fos. Another study revealed that PARP-1 expression gradually increases in nonatypical and atypical endometrial hyperplasia compared with normal endometrial epithelium (Ghabreau *et al*, 2004). Published data indicate that increased proliferation is a major determinant of PARP-1 expression and activity. Indeed, we cannot exclude the possibility that the lower PARP-1 protein and activity seen in the immortalised non-cancer HS-5 cells was merely due to the fact that this was also relatively slow growing cell line in our panel. Similar observation can be made for two slow growing cancer cell lines – Jurkat and ML-1 (doubling time 48 h) – which had very low PARP activity in our panel of cells. However, TR14 and Pre B697 cells were also slow growing (doubling time 72 and 30–40 h, respectively) but had high PARP-1 activity (both cell lines) and expression (Pre B697) so proliferation rate may contribute to, but not be the most important determinant of, PARP activity. Clearly, regulation of PARP-1 activity is complex and reflects phenotypic and behavioural changes within the cell that may make a greater contribution than either genotype or protein expression. Our study is the first to directly compare PARP-1 polymorphisms, cellular levels of PARP-1 protein and PARP activity in a systematic way. This reveals that PARP activity depends on other factors beside the level of protein and the active site SNP. Further studies to determine the factors that regulate the activity of this critical DNA repair enzyme are ongoing.

## Figures and Tables

**Table 1:** PARP-1 expression, activity and genotype in a panel of different cancer cell lines

**Cell line**	**(CA)_n_ genotype**	**T2444C genotype**	**PARP-1 expression**	**PARP activity**
Pre B697	SS	T/C	High	High
NGP	—	T/T	Medium	High
TR14	SS	T/T	Medium	High
NB1691	SS	T/T	High	Medium
SK-N-BE2C	SL	T/T	High	Medium
Nalm-6	SS	T/T	High	Medium
K562	SS	T/T	Medium	Medium
HL-60	SL	T/T	Medium	Medium
Molt-4	SL	T/T	Medium	Medium
MCF-7	SS	T/T	Medium	Medium
Raji	LL	T/T	Medium	Medium
LoVo	LL	T/C	Medium	Medium
LS	—	C/C	Medium	Medium
MDA-MB-231	SS	T/T	Low	Medium
T47D	SL	T/T	Low	Medium
ML-1	SL	T/T	High	Low
CCRF-CEM	SS	T/T	Medium	Low
TK6	SS	T/T	Medium	Low
Jurkat	SS	C/C	Medium	Low
HS-5	SS	T/T	Low	Low

Abbreviations: Promoter polymorphism SS–short alleles (CA)_11–12_/(CA)_11–12_, SL – short/long alleles (CA)_11–12_/(CA)_13–20_, LL – long/long alleles (CA)_13–20_/(CA)_13–20_; for active site T2444C SNP C/C is a variant/variant. Low-medium-high classification based on mean±s.d.
